# Adaptive UAV Attitude Estimation Employing Unscented Kalman Filter, FOAM and Low-Cost MEMS Sensors

**DOI:** 10.3390/s120709566

**Published:** 2012-05-21

**Authors:** Héctor García de Marina, Felipe Espinosa, Carlos Santos

**Affiliations:** 1 Discrete Technology & Production Automation Department, University of Groningen, Groningen, 9747 AG, The Netherlands; E-Mail: noeth3r@gmail.com; 2 Electronics Department, University of Alcala, Alcalá de Henares, Madrid 28871, Spain; E-Mail: carlos.santos@depeca.uah.es

**Keywords:** UAV navigation, attitude estimation, unscented Kalman filter, attitude heading reference system, fast optimal attitude matrix

## Abstract

Navigation employing low cost MicroElectroMechanical Systems (MEMS) sensors in Unmanned Aerial Vehicles (UAVs) is an uprising challenge. One important part of this navigation is the right estimation of the attitude angles. Most of the existent algorithms handle the sensor readings in a fixed way, leading to large errors in different mission stages like take-off aerobatic maneuvers. This paper presents an adaptive method to estimate these angles using off-the-shelf components. This paper introduces an Attitude Heading Reference System (AHRS) based on the Unscented Kalman Filter (UKF) using the Fast Optimal Attitude Matrix (FOAM) algorithm as the observation model. The performance of the method is assessed through simulations. Moreover, field experiments are presented using a real fixed-wing UAV. The proposed low cost solution, implemented in a microcontroller, shows a satisfactory real time performance.

## 1. Introduction

Nowadays the industry is employing UAVs for mobile missions, specially in vigilance, monitoring and inspection scenarios [1,2]. The stability and navigation of these autonomous vehicles need knowledge of its attitude angles [3,4]. Although these angles can be measured using a conventional Inertial Navigation System (INS), modern MEMS technologies are offering light and low cost solutions, which are more appropriate for the reduced space available for embedded systems in lightweight UAVs. However, these sensors are less accurate and more noisy than traditional mechanical or optics solutions. These inaccuracies result from the large biases and scale factors due to the sensors themselves and environmental effects such as temperature or mechanical vibrations. Moreover, inertial MEMS sensors do not provide attitude measures directly and the information proffered by them has to be post-processed through data fusion techniques. For this sensor fusion, the common solution in the literature is the Kalman filter. For fitting the highly non-linear kinematics model related to the attitude, the UKF is potentially better solution than the Extended Kalman Filter (EKF). The UKF is a *sigma-point* filter based solution, which is currently gaining growing interest in the attitude estimation area. Space application works based on UKF are showing more robustness and accuracy than the EKF [5]. Although their computational cost is still higher than EKF, there are new sigma-points algorithms aiming to reduce this cost, making it comparable with EKF in attitude estimation [6].

Three attitude angles can be represented in the Direct Cosine Matrix (DCM), which can be observed employing vectorial algorithms solving the Wahba's problem [7], such as Singular Value Decomposition (SVD), Three Axis Attitude Determination (TRIAD) [8] or FOAM [9]. For instance, TRIAD has been applied for attitude estimation in lightweight UAVs in [10], using as observation vectors the accelerometer and magnetometer readings. However, due to environment factors or mission's stage, the trust in sensors has to be adapted in a flexible way according to the actual scenario, therefore the observation model has to be adaptive. This paper introduces FOAM as observation model, which allows to set weights explicitly to the sensor readings, presenting clear advantages in the attitude estimation comparing with previous works that employ TRIAD and other sensor fixed-trust observations models.

Using a quaternion formulation, which is a conventional way to deal with the attitude of aerial vehicles [11], the DCM terms can be easily handled. The quaternion approach is widely used in AHRSs because it avoids the gimbal lock problem. With respect to numerical stability, quaternions are easier to propagate than the angles themselves.

For systematic and non-risky work, a simulation environment has been developed. The core of the simulation is the X-Plane 9 simulator, which is certified by the Federal Aviation Administration (FAA) for subsonic terrestrial flight. For realism [12], perturbations in the form of high-frequency noise, sensor latencies, biases, scale factors and misalignment are considered in the simulator data.

**Figure 1.**
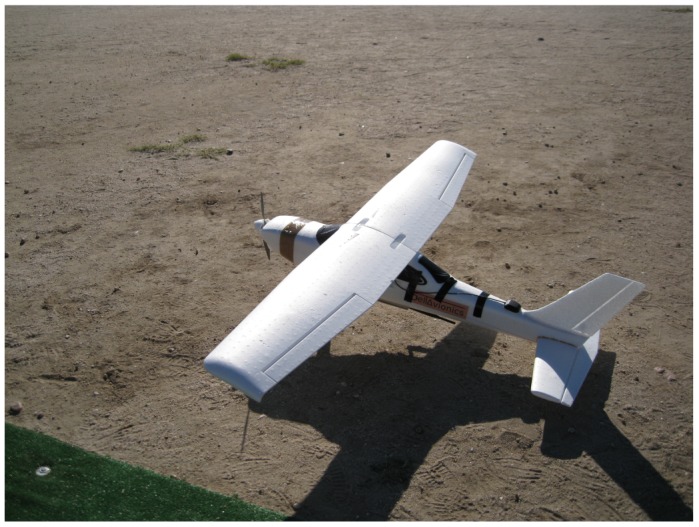
Mentor Multiplex aircraft modified by the authors to be a UAV.

During these simulations a maximum error of 1.0° is imposed on the pitch and roll attitude angles, and a maximum error of 4.0° on the yaw attitude angle. Under those requirements, which are standard in the industry [13], the error tolerances in the different signals are obtained through a Monte Carlo analysis. Finally, the algorithm is tested in a real experiment using a fixed-wing UAV with 2 meters wingspan. The modified aircraft to work as a UAV is the Mentor from Multiplex company. The UAV used in our experiments is shown in Figure 1. An in-house autopilot is used to test the estimation algorithm presented in this paper. The results of the solution are compared with different independent systems.

Summarising, the second section of the paper presents the state of the art about AHRSs. The following section is devoted to the formulation of the problem, including the kinematic model and how to adapt the observation model depending on the UAV situation using the FOAM algorithm. Then comes a section describing the whole solution using the UKF and FOAM. The fifth section deals with data obtained by simulation and it is compared with previous work where TRIAD algorithm was employed. Then, field experiments are presented to fully evaluate the proposed AHRS. In the final section of the paper, some conclusions are drawn.

## 2. Background and Related Work

The core of a well designed Kalman filter relies on an accurate model of the plant as well as the noise. Fortunately the attitude kinematics based on rate gyros is well known and corresponds to experimental results. However these equations are highly non-linear. This issue leads to the employment of the Unscented Transformation (UT) and the UKF, which have been introduced by Julier and Uhlmann [14]. Navigation algorithms employing this technique were further explained, with examples, by Wan and Merwe [15].

Most of the attitude estimation works trust in sensors in a fixed way throughout vehicle's mission [16], thus not allowing a real time adaptation depending on the actual environment factors, which is a common issue in small and lightweight UAVs.

A well-known problem with gyroscopes is the random walk bias which can not be identified at the calibration process. Nowadays, the random walk bias in gyroscopes based on MEMS technology can not be ignored even for short time missions. Therefore, different sensors have to be used to correct these random walk biases in real time. For instance, in [17] they are corrected using three-axis accelerometers. In [18] the use of eight accelerometers in a new configuration is proposed for measuring angular velocities in small UAVs.

Other approaches rely on magnetometers to estimate the yaw angle in helicopters [19]; or in Global Position System (GPS) to estimate both the position and the attitude in fixed-wing UAVs [20]. Alternatively, other papers propose not to use gyroscope at all in conventional aircrafts, but several GPS receivers instead [21].

The FOAM algorithm was introduced by Markley in [9] to measure the DCM of a spacecraft based on vectorial observations, but in this paper, it is used to estimate the quaternion components of the attitude. Actually, the TRIAD algorithm, introduced by Shuster and Oh in [8], is a particular solution of FOAM. With only two sensors available as sources, FOAM is light enough to be implemented in a microcontroller and a remarkable advantage is that it allows to tune the trust in the involved sensors through weights in execution time.

In the present paper, the authors propose an attitude estimator using the UKF and the FOAM algorithm as adaptive observation model. Gyroscopes, magnetometers and accelerometers based on MEMS technology are involved in the proposed estimator. The validation of the algorithm is done by both simulations and field experiments.

## 3. Problem Formulation

Despite the specific aerodynamic coefficients of the vehicle, it is possible to determine the attitude angles evolution from a kinematic model. In this section we derive the mathematical formulation of the AHRS problem in a UAV equipped with a three-axis gyroscope, a three-axis accelerometer and a three-axis magnetometer.

### 3.1. AHRS Kinematic Model

**Figure 2.**
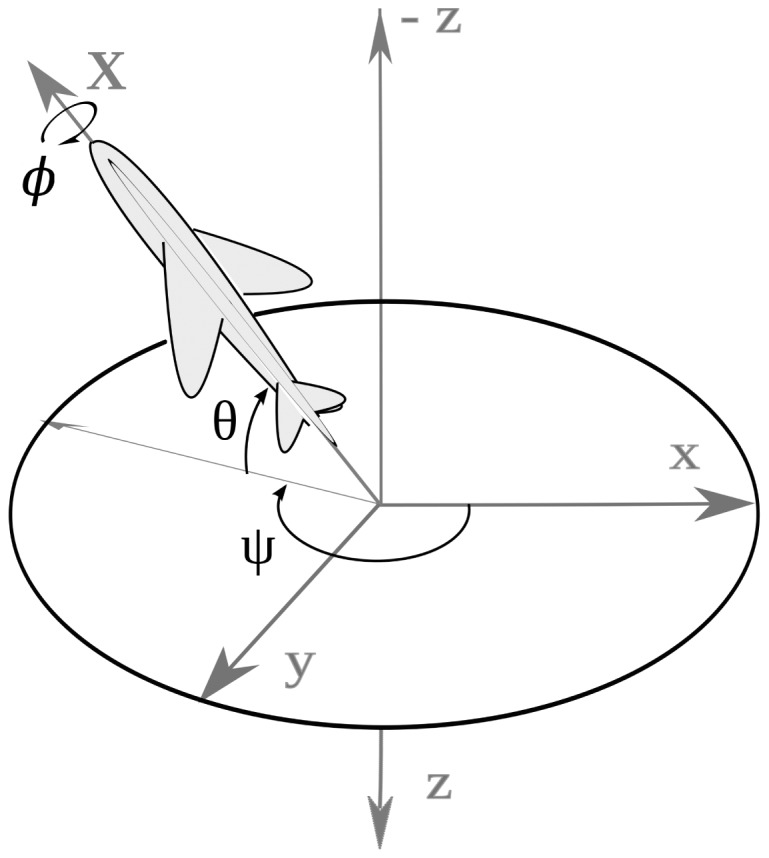
Axes and coordinate definitions.

The attitude angles describe the aircraft body-axis orientation in north, east, and down coordinates, *i.e.*, in longitudinal, lateral and normal coordinates, with respect to the local tangent plane to the Earth and true north. Here *θ* is the pitch angle, *φ* the roll angle and *ψ* is the yaw angle according to Figure 2. The angular velocity vector expressed in body frame includes three components: *P* the roll rate, *Q* the pitch rate and *R* the yaw rate; and it is related to the Earth frame by the transformation given by the kinematics Equation (1).

(1) $$\left[\begin{array}{c} \dot{\phi }\\ \dot{\theta }\\ \dot{\psi } \end{array}\right]=\left[\begin{array}{ccc} 1 & \tan\theta \sin\phi & \tan\theta \sin\phi \\ 0 & \cos\phi & -\sin\phi \\ 0 & \frac{\sin\phi }{\cos\theta } & \frac{\cos\phi }{\cos\theta } \end{array}\right]\;\left[\begin{array}{c} P\\ Q\\ R \end{array}\right]$$

Integrating Equation (1) gives numerical instability and could be gimbal locked. For this reason, a quaternion formulation to represent the attitude is preferred:

(2) $$\begin{array}{cc} q=q_{0}+q_{1}i+q_{2}i+q_{3}k & \sum_{i=0}^{3}q_{i}=1 \end{array}$$

where the quaternion norm is the unity and their components from attitude angles are:

(3) $$q_{0}=\cos\phi^{\prime }\cos\theta^{\prime }\cos\psi^{\prime }+\sin\phi^{\prime }\sin\theta \prime \sin\psi^{\prime }$$

(4) $$q_{1}=\sin\phi^{\prime }\cos\theta^{\prime }\cos\psi^{\prime }-\cos\phi^{\prime }\sin\theta^{\prime }\sin\psi^{\prime }$$

(5) $$q_{2}=\cos\phi^{\prime }\sin\theta^{\prime }\cos\psi^{\prime }+\sin\phi^{\prime }\cos\theta^{\prime }\sin\psi^{\prime }$$

(6) $$q_{3}=\cos\phi^{\prime }\cos\theta^{\prime }\sin\psi^{\prime }-\sin\phi^{\prime }\sin\theta^{\prime }\cos\psi^{\prime }$$

where *φ*′ = *φ*/2, *θ*′ = *θ*/2, and *ψ*′ = *ψ*/2.

The kinematics Equation (1) can be rewritten in linear form using quaternion components:

(7) $$\left[\begin{array}{c} {\dot{q}}_{0}\\ {\dot{q}}_{1}\\ {\dot{q}}_{2}\\ {\dot{q}}_{3} \end{array}\right]=\frac{1}{2}\left[\begin{array}{cccc} 0 & -P & -Q & -R\\ P & 0 & R & -Q\\ Q & -R & 0 & P\\ R & Q & -P & 0 \end{array}\right]\;\left[\begin{array}{c} q_{0}\\ q_{1}\\ q_{2}\\ q_{3} \end{array}\right]$$

The discrete formulation of Equation (7) has a closed form [22], this representation keeps the quaternion norm to the unity and it is numerical stable:

(8) $$q(k+1)=\left(I\cos\frac{\left\|\Delta \omega \right\|}{2}+\sin\frac{\left\|\Delta \omega \right\|}{2}\left\|\Delta \omega \right\|\Omega \right)q(k)$$

where $\left\|\Delta \omega \right\|=\frac{1}{2}\sqrt{{\left(P\Delta t\right)}^{2}+{\left(Q\Delta t\right)}^{2}+{\left(R\Delta t\right)}^{2}}$, *I* is the identity matrix, and

(9) $$\Omega =\frac{1}{2}\left[\begin{array}{cccc} 0 & -P & -Q & -R\\ P & 0 & R & -Q\\ Q & -R & 0 & P\\ R & Q & -P & 0 \end{array}\right]$$

Additionally, it is useful to formulate the DCM using quaternion components and the attitude angles from the DCM terms:

(10) $$\text{DCM}\equiv A=\{c_{ij}\}=\left[\begin{array}{ccc} A_{1} & 2(q_{1}q_{2}+q_{0}q_{3}) & 2(q_{1}q_{3}-q_{0}q_{2})\\ 2(q_{1}q_{2}-q_{0}q_{3}) & A_{2} & 2(q_{2}q_{3}+q_{0}q_{1})\\ 2(q_{1}q_{3}+q_{0}q_{2}) & 2(q_{2}q_{3}-q_{0}q_{1}) & A_{3} \end{array}\right]$$

where $A_{1}=q_{0}^{2}+q_{1}^{2}-q_{2}^{2}-q_{3}^{2},A_{2}=q_{0}^{2}-q_{1}^{2}+q_{2}^{2}-q_{3}^{2}$, and $A_{3}=q_{0}^{2}-q_{1}^{2}-q_{2}^{2}+q_{3}^{2}$. Then,

(11) $$\theta =-\arcsin(2(q_{1}q_{3}-q_{0}q_{2}))$$

(12) $$\phi =\text{atan}2(2(q_{2}q_{3}+q_{0}q_{1}),q_{0}^{2}-q_{1}^{2}-q_{2}^{2}+q_{3}^{2})$$

(13) $$\psi =\text{atan}2(2(q_{1}q_{2}+q_{0}q_{3}),q_{0}^{2}+q_{1}^{2}-q_{2}^{2}-q_{3}^{2})$$

where atan2 is the four-quadrant version of the inverse tangent function, and arcsin is the arcsine function.

### 3.2. Gyros Integration Problem

The three-axis gyroscope measures the angular velocities, and for obtaining the attitude angles, the gyros can be integrated using Equation (7). However, even if we ignore the sensor noise, the gyros suffer the random walk bias, increasing their integration error in every step.

Fortunately, for a MEMS gyroscope in normal conditions (not extremal temperature or pressure variation), this random walk varies really slowly, compared with the integration step, throughout the UAV mission. Therefore this bias for the gyroscope can be modeled as:

(14) $$\begin{array}{ccc} \dot{\text{b}}=0 & \text{with} & \text{b}={\left[\begin{array}{ccc} b_{x} & b_{y} & b_{z} \end{array}\right]}^{T} \end{array}$$

Denoting the angular velocity vector *ω* = [P Q R] *^T^*, if the angular velocities from the gyros are *ω_s_*, they can be corrected using the bias as:

(15) $$\omega =\omega_{s}-\text{b}$$

### 3.3. The FOAM Algorithm

The Wahba's problem [7] seeks for the optimal orthogonal matrix, which minimizes the next cost function:

(16) $$J(A)=\frac{1}{2}\sum_{k=1}^{N}a_{k}{\left|{\hat{W}}_{k}-A{\hat{V}}_{k}\right|}^{2}$$

where **W̃**_k_, *k* = 1… *N* is a set of *N* vectors measured with respect to body frame, **Ṽ**_k_, *k* = 1 … *N* is a set of *N* vectors with respect to the navigation frame, and *a_k_*, *k* = 1 … *N* are *N* positive weights.

The cost function Equation (16) can be rewritten as [23]:

(17) $$J(A)=\lambda_{0}-g(A)=\lambda_{0}-\text{tr}[AB^{T}]$$

where

(18) $$\begin{array}{ccc} \lambda_{0}=\sum_{k=1}^{N}a_{k} & \text{y} & B=\sum_{k=1}^{N}a_{k} \end{array}{\hat{W}}_{k}{\hat{V}}_{k}^{T}$$

and *tr* is the trace operator.

The two observations for the DCM determination in the UAV are the gravity vector and the Earth's magnetic field. The module of the gravity vector is the most stable, but it is not measurable directly; the accelerometers have to be compensated due to inertial effects and vibrations. The Earth's magnetic field is not affected by inertial effects, but it could be perturbed by standing close to the Earth's surface or environment as buildings, power lines, *etc.* Moreover, the Earth's magnetic field model does not have the same accuracy near of the Earth's surface than the gravity model. This is why it is necessary to modify the weights at Equation (16) throughout the flight. FOAM allows to tune these weights in run time.

The optimum *A* matrix in Equation (16) is based on the next characteristic equation [9]:

(19) $$0={\left(\lambda^{2}{\left\|B\right\|}_{F}^{2}\right)}^{2}-8\lambda \det B-4{\left\|\text{adj}B\right\|}_{F}^{2}$$

The attitude matrix is given by:

(20) $$A_{\text{opt}}={\left(k\lambda_{\max}-\det B\right)}^{-1}((B+\lambda_{\max}\text{adj}B^{T}-BB^{T}B)$$

where $k=\frac{1}{2}(\lambda_{\max}^{2}-{\left\|B\right\|}_{F}^{2})$.

If only two observations are available, the determinant of *B* is zero, simplifying the before expression to:

(21) $$\lambda_{\max}=\sqrt{a_{1}^{2}+a_{2}^{2}+2a_{1}a_{2}(({\text{w}}_{1}\cdot {\text{w}}_{2})({\text{v}}_{1}\cdot {\text{v}}_{2})+\left|{\text{w}}_{1}\times {\text{w}}_{2}\right|\left|{\text{v}}_{1}\times {\text{v}}_{2}\right|)}$$

(22) $$A_{\text{opt}}={\text{w}}_{3}{\text{v}}_{3}^{T}+(a_{1}/\lambda_{\max})({\text{w}}_{1}{\text{v}}_{1}^{T}+({\text{w}}_{1}\times {\text{w}}_{3}){\left({\text{v}}_{1}\times {\text{v}}_{3}\right)}^{T})+(a_{2}/\lambda_{\max})({\text{w}}_{2}{\text{v}}_{2}^{T}+({\text{w}}_{2}\times {\text{w}}_{3}){\left({\text{v}}_{2}\times {\text{v}}_{3}\right)}^{T})$$

where, w_3_ = (w_1_×w_2_)/|w_1_×w_2_|, v_3_ = (v_1_×v_2_)/|v_1_×v_2_|, w_1,2_ are the measured column vectors with respect to the body frame (accelerometers and magnetometers), and v_1,2_ are the two reference vectors in navigation frame (Earth's gravity and magnetic field model).

During a flight, sometimes the accelerometers are more reliable than magnetometers and vice versa. The criteria chosen to assign the weights are based on how close is the measured vector's module to the model vector's module. The measured magnetic field module can be perturbed by standing close to the Earth's surface, power lines, *etc.* The module readings from the accelerometer, once the centripetal effect had been compensated, will highlight if linear accelerations are present, such as a take-off or aerobatic maneuver. Considering these effects, the weights can be adapted by:

(23) $$\begin{array}{ll} & a_{k}=1-K_{k}\left|1-\frac{\left\|{\text{v}}_{k}\right\|}{\left\|{\text{w}}_{k}\right\|}\right|\\ \text{If} & \begin{array}{ccc} a_{k}\leq 0 & \text{then} & a_{k}=0.001 \end{array} \end{array}$$

where *K_k_* is a gain that indicates the weight sensibility due to the ratio between the module measured and modeled. This gain has been determined experimentally, showing a good behaviour of 1 for the magnetometer, and 2 for the accelerometer. This is because the accelerometer has to be compensated to obtain the gravity vector, while the magnetometer does not need any compensation.

The trust in the two sensors are related between their weights, so we can establish a weight maximum value of 1, and a minimum value of 0.001. Weights must be always positive and different from zero. In the last case, the FOAM algorithm is reduced to the particular case of TRIAD.

## 4. Unscented Kalman Filter as Sensor Fusion Core

The sensor fusion algorithm is implemented as a two-step *propagator/corrector* filter. It is desirable to run each step as many times as possible, however, the frequencies at which they are going to run are limited by different factors. For the propagation step, the limiting factor is the computation time; we chose a frequency of 100 Hz. This frequency is large enough for our experiments, since we do not expect angular velocities larger than 300 deg/s.

The criteria for selecting the frequency for the correction step are based on the random walk bias of our MEMS gyro, which is 0.007 deg/s (1*σ*). Assuming that we know perfectly the bias at the beginning, it takes about 9.75 s for diverging 1 degree from the true value. Assuming that the bias is always growing, which could be possible, for the worst case condition (taking 3*σ* for the random walk) it is obtained:

(24) $$\frac{3\times 0.007\deg/\text{s}}{2}x^{2}=1\deg\to x=9.75\text{s}$$

**Figure 3.**
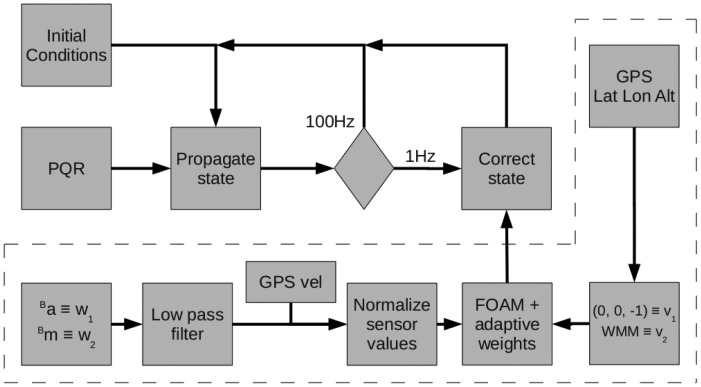
Algorithm block diagram. WMM is the World Magnetic Model and the GPS velocity is employed for subtract the centripetal acceleration.

In our case, the correction step is limited by the GPS velocity with respect to ground at 1 Hz sample rate, which fits safely with the before requirement. This GPS velocity is employed to compensate the centripetal acceleration in the accelerometers readings. The diagram of the algorithm is described in Figure 3. It can be noted how the FOAM information feeds the correction step. The area surrounded by dashed lines contains the elements involved in the FOAM calculations. The leftmost block represents an access to the sensors to measure *^b^a* and *^b^m*, which correspond to the accelerations and magnetic field measurements in the body reference frame respectively. The signals are then filtered and the GPS velocity is used to subtract the centripetal accelerations. On the right side of the diagram, the block denoted as *World Magnetic Model* uses an harmonic spherical model to obtain the magnetic field vector at the position of the UAV. This vector is used as one of the references in the FOAM algorithm. The references vectors are normalized, thus the model of the gravity reference vector is [0 0 −1]*^T^*

This section describes the design of the filter under the considerations given above. Subsection 4.1 details the equations involved in the propagation loop whilst Subsection 4.2 deals with those of the correction loop.

### 4.1. State Vector and Process Model

The state vector selected for the UKF is:

(25) $$x(k)={\left[\begin{array}{lllllll} q_{0}(k) & q_{1}(k) & q_{2}(k) & q_{3}(k) & b_{x}(k) & b_{y}(k) & b_{z}(k) \end{array}\right]}^{T}$$

where *q_i_* are the quaternion components and *b_j_* are the gyroscopes' biases. These are assumed to be Gaussian Random Variables (GRVs). Their process model is given by Equations (8),(14), respectively. This model identifies the gyroscopes' biases while propagates the vehicle's attitude compensating the gyroscopes readings. The detailed description of the UKF at its propagation stage can be found in [10,15].

The discrete-time Equation (8) assumes that the angular velocities remain constant during the discretization period. Hence, its process noise covariance matrix should be close to zero (but not zero). For the simulation and experimental results shown in this paper *Q_q_* = 1 × 10^−6^ · *I*_4_*_x_*_4_, where *I*_4×4_ is the 4 × 4 identity matrix, and subscript *q* refers to the quaternion model.

The process noise covariance associated to the gyroscope biases *Q_b_* is the 3 × 3 zero matrix. The rationale for this is the same that explains Equation (14) in Subsection 3.2.

### 4.2. Correction Equations and Observation Model

Even though the FOAM algorithm gives the nine terms of the DCM (see Equation (10)), only four of them are needed to calculate the attitude angles. Hence, the observation function has been designed to measure these four terms.

To determine the pitch and roll angles, the terms are the X and Y components of the Z earth vector expressed in the body frame:

(26) ZbEx≡c13=2(q1q3-q0q2)

(27) ZbEy≡c23=2(q2q3+q0q1)

For the yaw angle, the terms are the X and Y components of the X body vector expressed in the Earth reference frame:

(28) XbxE≡c11=(q02+q12-q22-q32)

(29) XbyE≡c12=2(q1q2+q0q3)

Therefore, the observation model is given by the following Equation:

(30) $$H(x_{k})={\left[\begin{array}{cccc} c_{13} & c_{23} & c_{11} & c_{12} \end{array}\right]}^{T}$$

According to the FOAM algorithm two vector pairs are needed to compute the terms of the DCM. Each pair consists of a measure or observation and a reference vector. In our case, these pairs are the magnetic field, and the acceleration of the UAV. The value of the measurement noise covariance can be derived from the nominal values of the involved sensor errors. This derivation is described thoroughly in [8].

As in the propagation stage, the detailed UKF correction stage can be found in [10,15].

## 5. Simulations Results

**Figure 4.**
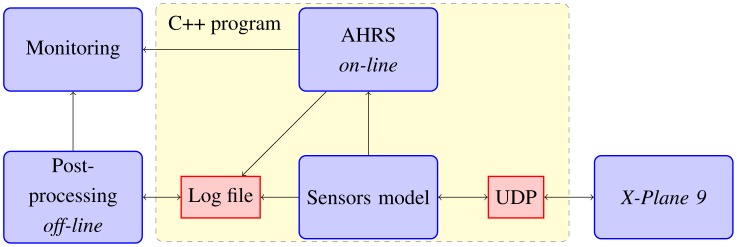
Block diagram of the simulation environment.

Since real experiments might imply crashes, some previous simulations have been carried out. For this, we developed a simulation framework, which consists of four different parts. The core of the simulation is the X-Plane 9 software. The other three parts are: the plug-in code for X-Plane 9, the realistic model of the sensors, and finally, the *online or off-line* processing of the sensors with the algorithm. The idea is to integrate a six-degrees-of-freedom aerodynamic model, provided by X-Plane with a realistic model of the sensors we are using. The plug-in code is just an UDP interface to link each other. Figure 4 shows the diagram block implemented to the *on-line* processing and for the post-processing.

X-Plane 9 includes different aircraft models. Its default radio control model is very similar to our UAV, so no additional modifications are needed. This is the model used in the simulations of this section. Because the airplane model is manually piloted, the navigation is not in any closed-loop; therefore if a tuning process is required, it could be done *off-line* with any software tool such as SciLab or Matlab. Also, the interface can be easily modified for Hardware in the Loop purposes, the class responsible for logging only has to write to a Serial Port instead of a file.

The purpose of the simulations is to study the effects of sensor noise, bias, latencies and filter convergence. Therefore, the model of the sensors focuses on these aspects:

- The GPS signal is delayed 1 s.
- The signals from the gyroscopes are biased with angular random walk, and corrupted with white noise.
- Accelerometers are biased with angular random walk and corrupted with colored noise, focusing in high frequencies.
- Magnetometers are biased with angular random walk and corrupted with white noise.
- Filter initial conditions are far from the actual initial values.

A first simulation challenge is to study the tolerances of the AHRS to bias and noise magnitude. According to standard procedures for Guidance and Control in aeronautics, a maximum error of 1.0° in the estimation of pitch and roll angle, and 4.0° for yaw angle, are imposed. It is assumed that with this error it is possible to do closed-loop control. This is covered in the first part of this section. The second part assesses the performance of the UKF using real values for the biases and noises magnitude of our sensors in a scenario where aerobatics are performed.

### 5.1. Sensor Error Tolerances in the AHRS

A Monte Carlo analysis of the tolerances was made, supported by a batch of simulation experiments. Each of the experiments specifies different values of biases and noise magnitudes, which were drawn from a Gaussian distribution. The values are shown in Table 1 versus the biases and random errors present in the UAV sensors of our real experiments, which are ADIS16405 from Analog Devices for IMU and magnetometers, and Lassen IQ from Trimble for the GPS.

As it can be appreciated the algorithm is more sensitive to errors in *R* than it is to errors in *P* and *Q*. This is because the information in the yaw angle is only provided by one of the sensors, the magnetometer. In contrast, the information in pitch and roll angles are provided by the accelerometer and the magnetometer.

The results show that the attitude estimator algorithm tolerances are large enough to embrace the actual UAV sensors on board. Therefore, the algorithm is suitable for being used with MEMS sensors.

### 5.2. FOAM and TRIAD Comparison in an Aerobatics Simulation Taking Onboard MEMS Sensors Error Values

**Figure 5.**
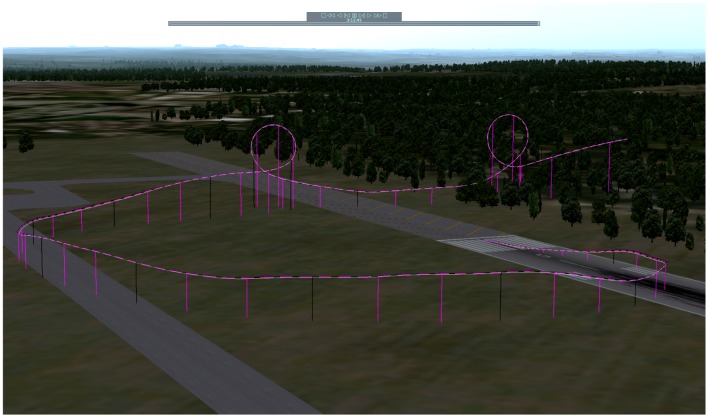
Aerobatics scenario with two *loopings*.

In this section we compare the FOAM algorithm against TRIAD in an aerobatics scenario. Figure 5 shows this scenario performing two loopings. The filter is reset just at take-off time, so there is not a stationary situation for helping to filter convergence.

For this simulation, the real biases and noise magnitudes were extracted from our sensors' data sheet and they can be consulted in Table 1.

**Figure 6.**
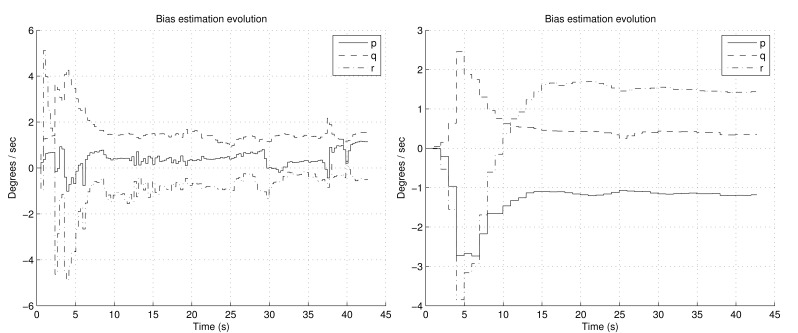
Estimation of the biases of the gyroscopes, employing TRIAD at the left and FOAM at the right during the aerobatics simulation shown in Figure 5.

**Figure 7.**
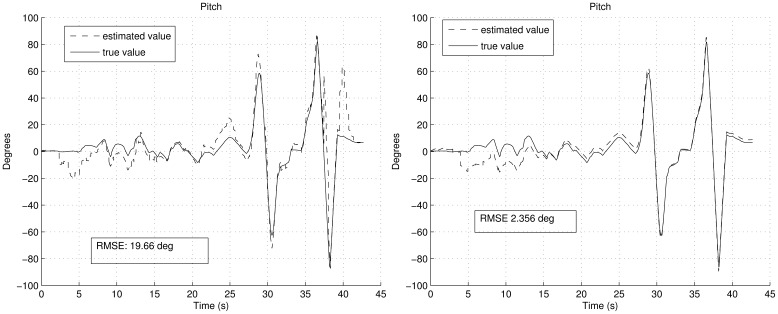
Pitch angle estimation during the aerobatics, employing TRIAD in the left and FOAM in the right during the aerobatics simulation shown in Figure 5.

**Figure 8.**
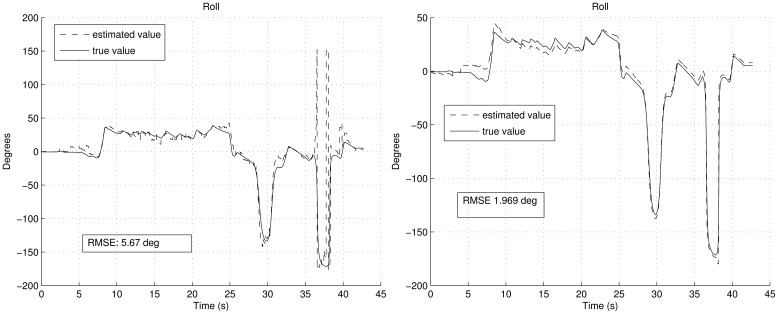
Roll angle estimation during the aerobatics, employing TRIAD in the left and FOAM in the right during the aerobatics simulation shown in Figure 5.

Figure 6 shows the estimation of the biases. It can be noted how the convergence is smoother with FOAM in the right graph than with TRIAD in the left graph. Moreover, since in TRIAD the accelerometers are always trusted in a fixed way, wrong accelerometers corrections lead to a wrong estimation of the biases, which finally gives a less confident estimation in the attitude angles as result. This is shown in Figures 7 and 8, after the convergence time (around the fifteenth second) in the left graphs, the true and estimated value do not converge properly due to the wrong gyroscopes' bias estimation. It can be noted that the right graphs have better RMSE, highlighting that the RMSE also includes the transitory period.

**Figure 9.**
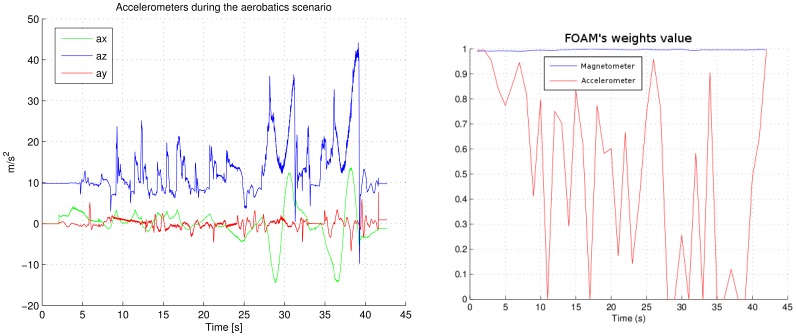
Accelerometers readings and evolution of the FOAM weights during the aerobatics simulation shown in Figure 5.

Right graph in Figure 9 shows the evolution of the FOAM's weights during the aerobatics. As it can be appreciated, accelerometer weight has a low value when an aerobatic is being performed. The aerobatic can be analyzed in the left graph in Figure 9, where the norm of the three accelerations are not the gravity value. This adaptation in weights avoids disturbing the estimation of the roll and pitch angles by the AHRS.

However, the final bias values even in FOAM are not stationary due to the random walk noise in gyroscopes. Nevertheless, it can be noted how this random walk evolution is slower than the system dynamics.

The RMSE shown in Figures 7 and 8 includes the convergence time. Despite the convergence time (from fifteenth second), using the UKF with the FOAM the RMSE is 0.974° and 0.873° respectively. Therefore, the established requirements in the earlier subsection are achieved, even in aerobatics scenario.

During the simulations, although it is not very clear in the graphs, it is observed that the proposed solution with FOAM does not introduce big delays.

## 6. Field Experiment Results

Two real experiments were carried out using the small fixed-wing UAV shown in Figure 1. The first one is a take off. This scenario is representative because the accelerometers are measuring not only the gravity but also the linear acceleration, vibrations due to the ground, lift forces, *etc*. The second experiment is a circular flight with the presence of wind gusts as perturbations.

**Figure 10.**
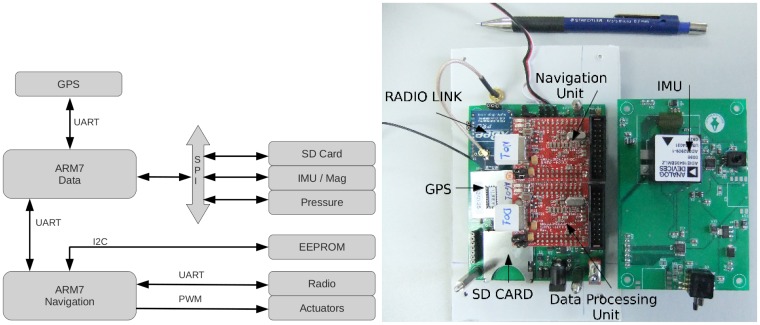
Functional diagram of the autopilot and its hardware implementation.

An on-board autopilot hardware has been designed and built for this UAV. Figure 10 shows a functional diagram of the autopilot and its hardware implementation.

Two ARM7 microcontrollers are assigned for sensor data handling and navigation algorithms. Both are connected with a UART channel. Flight data are measured by different sensors: a GPS receiver, an Inertial Measurement Unit (IMU) with magnetometers, and four pressure sensors. One of the pressure sensors is used as a barometer for altitude measurement and the rest, one per axis, are connected to Pitot tubes for air-speed measurement. Sensor data are stored in an SD card for experiment post-processing analysis.

The objective of the experiments is to assess the accuracy of the estimation of each of the three attitude angles during a non-stationary flight such as the take-off, and to assess the filter convergence during a long flight. A ground station was built to receive data from the UAV using a radio link. The flight is manually controlled using a conventional radio control unit.

For validation purposes, data coming from independent sensors (not used by the estimation algorithm) have been considered. For the roll angle, a computer vision system is used. The GPS velocity is used to validate the yaw angle.

**Figure 11.**
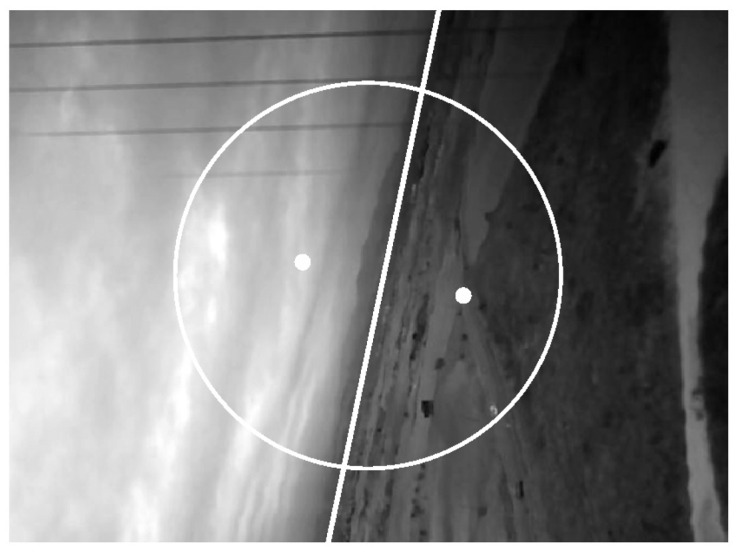
One of the frames processed by the vision system capturing the roll angle.

The vision system uses a small camera attached to the UAV. An algorithm was developed to obtain the roll angle from the video measuring the slope of the horizon. The algorithm is based on [24–26]. Figure 11 shows one of the frames taken during the flight and processed by the vision system. The results of this system have an uncertainty of ±4°. This systematic error comes from the statistical processing of the video frames; in this way, the roll angle can be determined directly by hand from the horizon.

**Figure 12.**
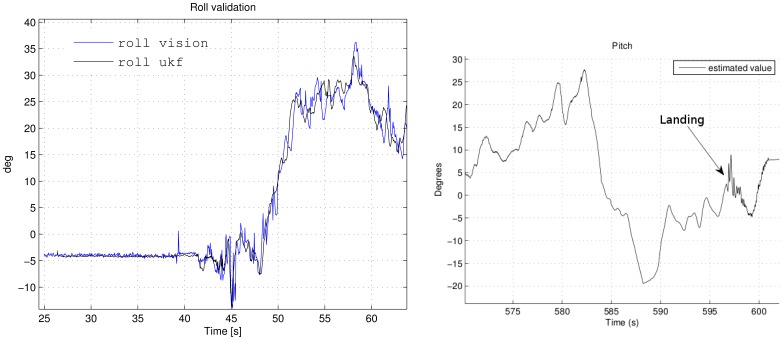
Roll angle comparison between vision system and UKF estimation and Pitch angle at the landing time after ten minutes of flight.

**Figure 13.**
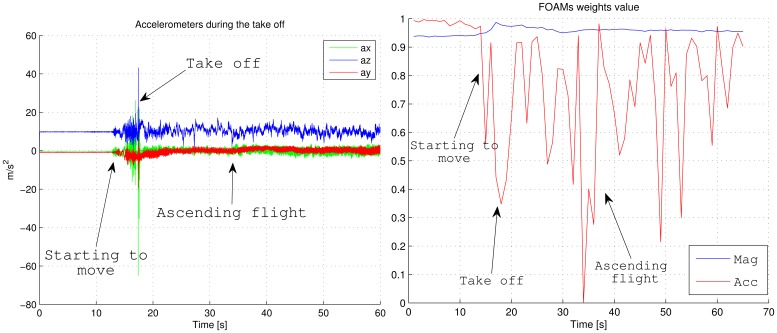
Accelerometers readings and FOAM weights during taking off.

The left graph in Figure 12 shows the roll angle evolution during the take off experiment. The vision algorithm indicates an initial roll angle of −5° due to the runway inclination, which coincides with the estimated by the algorithm. This scenario involves high accelerations and it is described in detail in Figure 13. The results of the comparison are clearly satisfactory, both algorithms follow the same tendency and magnitude order, highlighting that the roll estimation has not been affected by the presence of high accelerometer readings.

The right graph in Figure 12 shows the vehicle landing after ten minutes of flight. As it can be appreciated the AHRS integrity is held throughout the flight, estimating the same pitch value at the end of the flight compared with the start, which was closer to 7.5°. Thus, the biases have been estimated rightly.

Figure 13 shows the acceleration and the FOAM's weight evolution during the taking off maneuver. Although they describe the same take off maneuver in Figure 12, the time axis is biased with respect to the vision algorithm because both systems have been started at different times. Before the twelfth second, the UAV has been in a steady position where the accelerometers only measure the gravity. Then at the twelfth second, the vehicle begins to accelerate for reaching enough velocity for the take off. Note that all the vibrations are due to a rugged runway at this stage. At the seventeenth second the UAV pilot *pulls up* the elevator for lifting the vehicle, and at this stage the vehicle suffers a high acceleration maneuver, close to 4 g. We can see how the weights at the start are more confident when the accelerometer is only measuring the gravity (nonlinear acceleration is present), and the minimum peaks are reached at the *pull-up* stages during the take off and different ascending moments, due to the manual control of the vehicle. The weight related to the magnetometer differs from the modeled one due to the Earth's surface and power lines next to the runway. Once the UAV gains altitude, this weight becomes more confident. Therefore, the AHRS can better adapt for different situations than keeping the trust in sensors constant.

The second experiment took place under adverse atmospheric conditions, in particular, there were gusts of wind. So when there is strong crosswind, the yaw angle differs from the heading of the plane. Even if there is no wind, the plane slides due to its inertia when it turns. The heading is measured using the GPS velocity with an uncertainty of ±10°. It can be noted that the GPS velocity is only used to subtract the centripetal contribution of the accelerometers in the estimation algorithm. Therefore, it can be taken as an independent system for validation purposes.

**Figure 14.**
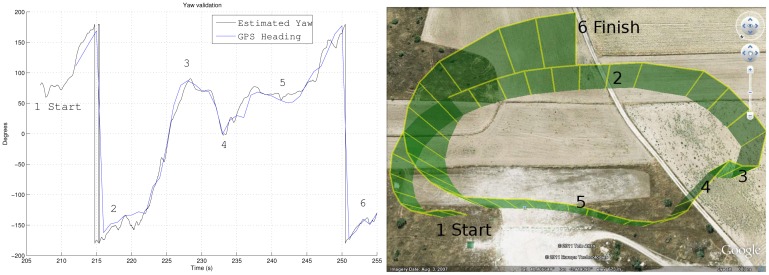
Yaw angle comparison between GPS system and UKF estimation.

Figure 14 shows a comparison of the yaw angle estimated and the heading measured by the GPS. The figure corresponds to a one and a half turn, and the different stages are marked with numbers in each figure. It can be noted how the North points to the bottom left corner.

During the area marked with “1”, the UAV faces the wind, therefore the GPS footprints are closer each other. The area marked with “2” shows the effect of crosswind; it biases the yaw angle and the heading, which is known as to have a non-zero *side slip* angle. In other words, the UAV does not move in the direction it points to. In the area marked with “4”, the end of the first turn appears closer to 0° (pointing to the North) in heading and yaw angle. Finally, there is a clear correspondence between the GPS readings and the algorithm estimation, following the same tendency during the flight.

The sensors used in the field experiments do not allow to have an independent measurement for the pitch angle validation. However, due to the formulation of the algorithm using quaternions, the pitch angle is strongly coupled to the roll and yaw angles. Therefore, it can be assumed that if the roll and yaw angles are both correctly estimated, the pitch angle is correctly estimated too.

## 7. Conclusions

This paper considered the navigation problem of attitude estimation of a light-weight UAV. Due to the severely limited free space of this kind of vehicles, MEMS accelerometers and gyroscopes are only suitable for being on board.

Previous studies in the attitude determination context give the same confidence to the sensors throughout the whole UAV mission. This is unsuitable for missions that mix both aerobatic and non-aerobatic maneuvers. A common solution in the satellite attitude estimation is the FOAM algorithm and it has been used as the observation model in the UKF framework due to the highly non-linear kinematic model of aircraft attitude. An adaptive algorithm to determine the weights in FOAM algorithm has been presented, showing reliable results of the attitude along the different phases of a flight.

The performance of the algorithm has been validated by simulation and assessed using field experiments. By means of independent sensors it has been checked that the estimation algorithm gives good results, confirming the suitable quality of the estimations. The algorithm is light enough to be run on an onboard system with minimal electronic resources. The experimental results give confidence to pursue next works concerning automatic control implementation.

## Figures and Tables

**Table 1.** UKF tolerances and actual onboard sensors: Maximum bias and random error standard deviation. It can be noted how the algorithm based on UKF meets the onboard values.

**Measurement**	**UKF tolerance**	**Sensor Bias error**	**UKF tolerance**	**Sensor Random Error**
Roll rate, P	±11.38°/s	±3.00°/s	±8.82°/s	±1.00°/s
Pitch rate, Q	±11.38°/s	±3.00°/s	±8.82°/s	±1.00°/s
Yaw rate, R	±11.38°/s	±3.00°/s	±5.32°/s	±1.00°/s
Accelerometers	±0.3 m/s^2^	±0.05 m/s^2^	±0.7 m/s^2^	±0.009 m/s^2^
Magnetometers	±10.63 mG	±4.00 mG	±22.53 mG	±1.25 mG
GPS Velocity	±2.46 m/s	±0.5 m/s	±2.35 m/s	±1.5 m/s
